# Barnes on Diseases of Women

**Published:** 1874-04

**Authors:** 


A Clinical History of the Medical and Surgical Diseases of Women. By Robert Barnes, M.D., London, etc., with 169 illustrations. Philadelphia: Henry C. Lea, 1874. Octavo, pp. 792. Leather, $6.

The announcement of a new work upon the diseases of women by Robert Barnes, of London, is calculated to raise the professional mind to the tip-toe of expectancy. While a cursory survey of the shelves of the publishers finds no paucity of modern works, and good ones too, upon the diseases of women, we cannot esteem this of Dr. Barnes either redundant or untimely. One need only turn over the pages of the standard treatises upon this specialty to find the widest diversity of opinion and advice, emanating from gentlemen of high authority in the profession, and this, too, upon almost every important topic discussed. One has only to consult his own observation of his cases in practice, if he will but follow them carefully for a few years, to learn how disappointing and unsatisfactory is, for the most part, the remedial measures inculcated. A reflecting mind cannot doubt that the whole subject of pathology and treatment of uterine and ovarian lesions, although rapidly advancing, has, as yet, reached little that is fixed and certain. It is out of the *multitude of counsel*, coupled with rigid analysis of our daily experience, that we must learn. We doubt if it be proper, in the present state of the science and art, to put into the hands of a gynæcological student any existing work as a text-book invested with *authority*. Quite certain are we that no one can *practice* gynæcology with satisfaction and success who relies upon the dictum of any one author. Heartily, then, do we welcome new light upon this dark and difficult subject from one who has so good right to teach as Robert Barnes, of London.

In a hasty review of Dr. Barnes' work, we observe that he regards the ovary as the "primum mobile of menstruation." He cites the celebrated case of Percival Pott as authority, and, like Leishman of Glasgow, makes no mention of the counter results obtained in this country by Atlee, Storer, Jackson and the writer, as well as Meadows, in his own city of London.

Under the head of amenorrhœa, the case of J. C———, aged eighteen, in St. George's Hospital,* is quoted, and the author remarks: "There being no uterus, the menstrual blood sought outlet in every direction, and the function failing, the patient died. * * Possibly an operation for the construction of a vagina to open a communication with the rudimentary uterus might have been of service." Evidently, the thought of removing the *exciting cause* of the fatal vascular and nervous purturbation was not in his mind. In speaking of hernia of the ovary, our author advises "if the ovary be fixed by adhesions, it may be wise to follow the example of Pott." How much *wiser* would it have been to have rescued the life of J. C——— by a similar proceeding.

The author gives the credit of the first successful ovariotomy to McDowell, and his treatment of the subject of ovarian tumors and ovariotomy is concise, clear and judicious. In catarrha endo-metritis the employment of intra-uterine injections is fully discussed, their remedial value set forth, and their great danger properly pointed out. "The general conclusion at which I have arrived is to restrict the use of intra-uterine injections within the narrowest limits. I rarely employ them now, except in cases of urgent danger from metror-rhagia." Again: "One all-important caution is to be religiously observed, namely: *never to use any topical application to the uterus, or to perform any surgical operation upon the uterus, when a menstrual period is impending.*" Dr. Barnes thinks well of pessaries, and especially of Hodge's lever or bow pessary.

Of cancer of the uterus our author believes, in a certain class of cases, complete cure by operation is *not* hopeless, and in the majority of cases operative measures serve to prolong life, and add greatly to the comfort of the patient.

The volume is gotten up in handsome style, the typography excellent, the illustrations numerous and good. We predict for it a large sale.

---

THE BRITISH JOURNAL OF DENTAL SCIENCE. *Established sixteen years.* A monthly chronicle of current intelligence, relating to every Department of Dentistry and the collateral sciences, published on the first of each month.

It may be had of the American Agents, Lindsay & Blakiston, Philadelphia, at $4.00 per year, or 50 cents a copy.

---

* Transactions Med. Assoc. Georgia, 1873, p. 57.

